# Why does FND mainly affect women? A consideration of gender imbalance in neuropsychiatric disease

**DOI:** 10.1017/S0033291726104085

**Published:** 2026-04-13

**Authors:** Richard Bradlow, Laure Von Der Weid, Sav Zwickl, Ada Cheung, Richard Kanaan

**Affiliations:** 1 Department of Psychiatry, University of Melbourne, Austin Health, Heidelberg, Australia; 2 University of Freiburg Faculty of Medicine: Albert-Ludwigs-Universitat Freiburg, Germany; 3 Trans Health Research Group, Department of Medicine, The University of Melbourne, Heidelberg, Australia

**Keywords:** functional neurological disorders, Gender, neurological illnesses, psychiatric illnesses

## Abstract

**Background:**

Functional neurological disorder (FND) is a common and disabling neuropsychiatric condition in which women comprise approximately 75% of cases. This paper examines whether the gender imbalance seen in FND is unique among neurological and psychiatric conditions and explores the biological, psychological, and social contributors to this disparity.

**Methods:**

A narrative review was conducted using MEDLINE, PsycINFO, and Web of Science. Gender ratios were compared across depression, anxiety, post-traumatic stress disorder, schizophrenia, eating disorders, Parkinson’s disease, and multiple sclerosis. Evidence regarding sex hormones, early life trauma, gender-based social determinants, and diagnostic biases were synthesized thematically.

**Results:**

Amongst the psychiatric and neurological conditions reviewed, FND shows a pronounced female predominance (approximately 3:1), placing it amongst the most gender imbalanced disorders in our sample, with only eating disorders showing a larger female predominance. Biological factors (particularly the influence of estrogen and progesterone on stress reactivity, neuronal excitability, and agency), may heighten female vulnerability. Social determinants (increased exposure to trauma, socioeconomic inequality, and gender norms) further contribute to this risk. FND shares clinical and demographic similarities with other internalizing disorders and conditions linked to dissociation and trauma. The literature suggests FND emerges from a bidirectional interaction between gonadal hormones and psychosocial stressors.

**Conclusions:**

The marked gender imbalance in FND arises from the interplay of biological vulnerability and gendered social adversity. Understanding these intersecting influences is essential for reducing stigma and guiding future research, diagnosis, and treatment. The findings support the need for a gender-sensitive, biopsychosocial approach to FND care, and investigation.

## Introduction

Functional neurological disorder (FND) is a neuropsychiatric condition characterized by neurological symptoms causing significant disability, but which have no neuropathological explanation (AMA, 2013). It is common, particularly in younger people(Hallett, Stone, & Carson, 2016; Tinazzi, Gandolfi, Landi, & Leardini, 2021), with a prevalence rate of approximately 50/100,000, (Aybek & Perez, 2022) though this is likely an underestimate as many individuals with FND remain undiagnosed for years(Crimlisk et al., 2000). FND causes significant functional impairment, distress, stigma, and reduced quality of life (Carson et al., 2011; Tinazzi et al., 2021).

For much of human history, FND was considered a gynecological disorder (“hysteria”), and so exclusively to affect women (Veith, 1993). Whilst this was obviously a fallacy, FND remains far more common in women than men (Morsy et al., 2021), despite it now being considered a neuropsychiatric disorder. It continues to be uniquely stigmatized as essentially a disorder of women (McLoughlin et al., 2023), with the diagnosis itself seen by many as an expression of misogyny (Micale, 1995).

The reasons behind this gender imbalance are unknown, with biological, psychological, and social explanations suggested (Chouksey & Pandey, 2020). Biologically, sex hormones might contribute to the imbalance (Martel, 2013), which is supported by the female preponderance increasing substantially after menarche and reducing after menopause (Asadi-Pooya & Homayoun, 2020; Baizabal-Carvallo & Jankovic, 2020; Campo, Jansen-McWilliams, Comer, & Kelleher, 1999; Lidstone, Costa-Parke, Robinson, Ercoli, & Stone, 2022). Psychologically, the gender imbalance might be secondary to a differential response to early life trauma. For example, childhood sexual abuse is more commonly experienced by girls and appears to more strongly increase their risk of developing FND than boys (Kletenik et al., 2020). Socially, it might be a response to the place of women in society, with the life events commonly triggering FND particularly impacting women (Ludwig et al., 2018; Morsy et al., 2021). The research into any of these is minimal, however, and the gender imbalance remains ultimately unexplained.

Gender differences exist in many illnesses, however, and some may be far better understood than FND. In this paper, we compare FND with other neuropsychiatric disorders to identify whether the gender ratio in FND is unique, and which psychosocial/biological factors are most implicated in the gender divide. Whilst we present psychiatric and neurological conditions in separate sections, we acknowledge that this an artificial dichotomy and use it to emphasize that FND sits at the intersection of these domains as a neuropsychiatric disorder. We argue that the pronounced gender disparity in FND arises through the complex interplay of biopsychosocial factors, with particular emphasis on the intersectionality between hormonal influences, exposure to traumatic events, and social determinants shaped by gender norms.

## Sex and gender

One challenge to disentangling this complexity is the conflation of sex and gender. *Gender*, in this paper, refers to the role defined by social, psychological, cultural, and behavioral factors, and *sex* is determined by anatomical and physiological markers (Muehlenhard & Peterson, 2011). However, the research this paper references for FND mostly did not distinguish between sex and gender, and there is no way we can know how many participants had a gender that differed to the one assigned from birth. In this paper, *gender* will be used unless explicitly discussing physiological processes. Similarly, the vast majority of the literature refers to only two genders, even though we would today consider there to be many (Lindqvist, Sendén, & Renström, 2021). This paper will focus on comparing the man/woman gender ratio only but acknowledges there are multiple genders and that people who are gender diverse are unequally affected by illness (Abel, Drake, & Goldstein, 2010).

Previous research has also largely ignored, or not reported on, the presence of trans people in FND patient populations, though research that has considered it has found trans people to be overrepresented in FND (Bradlow, Meyer, & Kanaan, 2024; Lerario, Rosendale, Waugh, Turban, & Maschi, 2023). Papers that have reported on the presence of trans people in FND patient populations have found that the cisgender gender ratio has remained the same (3:1 women to men), implying that the both the sex and gender ratio of FND is 3:1 (Bradlow et al., 2024). This is likely because the overall number of FND patients who are trans is likely too small to significantly affect the gender ratio.

## Methods

This article is a narrative review that aims to contextualize and explain the gender ratio in FND by comparing sex/gender ratios across selected neurological and psychiatric conditions. We conducted a systematic search for each condition but did not undertake other systematic review procedures (e.g. PRISMA workflow, dual screening, or formal risk-of-bias appraisal). Rather, we then conducted a narrative thematic synthesis of proposed mechanisms that may contribute to female predominance, organizing findings into four domains (sex hormones, early life trauma, gender-based social determinants, and diagnostic bias), and refining these themes during manuscript development.

Searches were performed on OVID (MEDLINE), PsycINFO, and Web of Science using the search term ‘gender ratio’ combined with the following specific illness terms (autism, substance use disorder, schizophrenia, bipolar affective disorder, anxiety disorder, obsessive compulsive disorder, post-traumatic stress disorder, depression, eating disorder, functional neurological disorder, Parkinson’s, epilepsy, migraine, Alzheimer’s, and multiple sclerosis). Synonyms for conditions were also used in searches.

Conditions were chosen to represent a spectrum of psychiatric and neurological illnesses with recognized gender differences.

In addition to the search for gender ratios, we undertook a review of biological, psychological, and social contributors to gender differences in neuropsychiatric conditions. This process was guided by known determinants of sex and gender disparities identified in prior reviews, including hormonal influences, early life trauma, gender-based social determinants, and diagnostic biases. Reference lists of key papers were also examined to identify additional relevant literature. The purpose of this broader review was to synthesize evidence on mechanisms that may contribute to the gender imbalance observed in FND.

All searches were conducted by RB from 10/05/24–31/08/24. Interpretations were discussed among all authors.

## Gender ratio in psychiatric conditions

**Figure 1.**
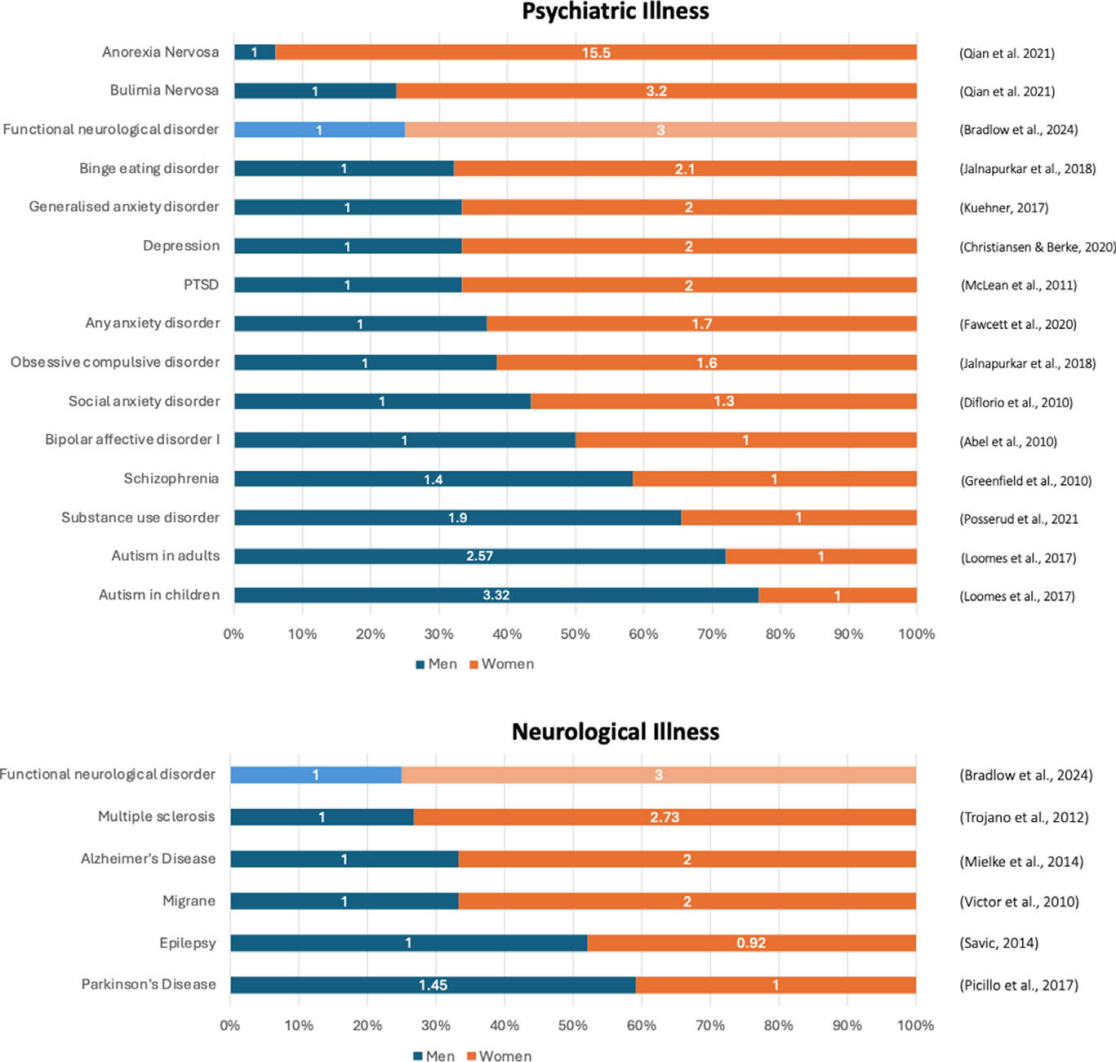
Gender difference in psychiatric and neurological illnesses.

Gender differences in psychiatric disorders and neurodevelopmental conditions are well-documented, with some illnesses, such as schizophrenia and autism, predominantly affecting men (Abel et al., 2010; Posserud, Skretting Solberg, Engeland, Haavik, & Klungsoyr, 2021, and others, such as post-traumatic stress disorder (PTSD), depression, and eating disorders, predominantly affecting women (Christiansen & Berke, 2020; Kuehner, 2017; Qian et al., 2022) (Figure 1).

In the context of other psychiatric conditions, the gender ratio seen in FND (3:1) (Bradlow et al., 2024) is large but not an outlier. It is notable that the illnesses that for some are considered more attributable to psychosocial causes, such as eating disorders and FND, have a more pronounced gender ratio (Culbert, Sisk, & Klump, 2021; Melisse, de Beurs, & van Furth, 2020; Qian et al., 2022).

## Gender ratio in neurological conditions

Like psychiatric conditions, neurological conditions also have well documented gender differences. Parkinson’s disease (PD) and epilepsy predominantly affect men (Picillo et al., 2017; Savic, 2014), whilst multiple sclerosis (MS) and Alzheimer’s disease (AD) predominantly affect women (Mielke, Vemuri, & Rocca, 2014; Trojano et al., 2012). Compared to psychiatric conditions, however, these ratios tend to be smaller. The reasons for these gender differences remain unclear. In the context of neurological conditions, the gender ratio seen in FND (Bradlow et al., 2024) appears larger than that observed in other neurological conditions.

## Explanations for gender differences seen in psychiatric conditions

### Biological factors

Sex hormones have been clearly implicated in several psychiatric disorders, with multiple mechanisms of action suggested. Estrogens and progesterone can affect the brain epigenetically or directly. They easily cross the blood brain barrier, have similar concentrations in the blood and brain, and influence the central nervous system through interaction between receptors and ligands, alteration of conductance of ion channels through allosteric modulation, and increase in neuron excitability (Allais et al., 2020). Epigenetically, they can modulate the production and metabolization of multiple neurotransmitters including calcitonin gene–related peptide (CGRP), serotonin, glutamate, noradrenalin, nitric oxide, and endogenous opioids (Allais et al., 2020). The hypothalamic–pituitary–adrenal (HPA) axis, which regulates stress responses, and the hypothalamic–pituitary-gonadal (HPG) axis, which regulates sex hormones, interact in a bidirectional manner (Mu, Thomas, & Kulkarni, 2022). This occurs through altered gamma-aminobutyric acid modulated regulation of the HPA axis (Kuehner, 2017).

There is ample evidence that fluctuations in estrogen and progesterone increase the risk of someone developing a depressive or anxiety disorder, or PTSD (Christiansen & Berke, 2020; Kuehner, 2017). Higher estradiol levels also increase serotonergic neurotransmission (Karpinski, Mattina, & Steiner, 2017). Testosterone has been found to be an anxiolytic, possibly through reducing HPA axis reactivity (Christiansen & Berke, 2020). Estrogen can also be an anxiolytic, but its effect appears to be dose-dependent, in an inverted ‘U’ shape, where too much or too little estrogen can exacerbate anxiety (Maeng & Milad, 2015). These same mechanisms may be relevant to FND, given that altered stress reactivity and heightened neuronal excitability are implicated in functional seizures and other FND symptoms (Keynejad et al., 2019).

The timing of the gonadal hormone exposure mediates the effect. In males, higher testosterone exposure, prenatally and pubertally, appears to be protective of developing an eating disorder (Culbert et al., 2021). In females, lack of early testosterone exposure and lower estradiol, prenatally and pubertally, increase risk of developing an eating disorder (Culbert et al., 2021). However, in females, higher testosterone levels may increase the risk of developing an eating disorder, which differs to its protective effect in men (Culbert et al., 2021). Sex hormones, with genetic factors, are believed to mediate the gender differences seen in brain development, prenatally and during puberty (Abel et al., 2010. These differences contribute to females having a lower risk of developing schizophrenia and a higher risk of developing depression (Abel et al., 2010; Kuehner, 2017).

A wide range of psychiatric conditions are affected by stages of the menstrual cycle, pregnancy or menopause. This includes premenstrual dysphoric disorder, postpartum depression, perimenopausal depression (Kuehner, 2017) but also obsessive-compulsive disorder (OCD) (Karpinski et al., 2017) (Green & Graham, 2022), PTSD (Green & Graham, 2022; Mu et al., 2022), schizophrenia (Abel et al., 2010, and anxiety disorders (Green & Graham, 2022; Kuehner, 2017). Mood episodes of bipolar can be influenced by pregnancy, the postpartum period, and different stages of the menstrual cycle (Rusner, Berg, & Begley, 2016; Teatero, Mazmanian, & Sharma, 2014). Tamoxifen, a selective estrogen receptor modulator (SORM), is emerging as a treatment of mania and has been found to increase the likelihood of developing depression (Carmassi et al., 2021), this implies that estrogen or fluctuations in estrogen may have a causative role in mania or be preventative of bipolar depression. Females with schizophrenia are more likely to have low estrogen levels and irregular menstrual cycles and more likely to have severe symptoms during low estrogen stages of their menstrual cycle (Abel et al., 2010).

Sex hormones and the menstrual cycle affect how someone responses to stressors potentially leading to the development of a trauma disorder such as PTSD. PTSD symptoms fluctuate in severity during the menstrual cycle with relieving symptoms being the most severe when estrogen and progesterone levels are high, and mood and phobic symptoms being strongest when estrogen levels are low (Mu et al., 2022). On magnetic resonance imaging studies, males and females have been demonstrated to have similar stress responses when females are in a low estrogen phase of their menstrual cycle, however men have significantly higher increases in blood flow and volume within the hippocampus, orbitofrontal cortex, medial prefrontal cortex, lateral hypothalamic area, left amygdala, and anterior cingulate gyrus (a key brain region in stress response, anxiety disorders, and fear) compared to females in high estrogen phase of their menstrual cycle (Maeng & Milad, 2015). When stressed, males display reduced fear conditioning compared to females, which correlates with impaired fear responses in the anterior cingulate cortex and amygdala, while females display enhanced fear learning and a correlated different response in the anterior cingulate cortex and amygdala (Maeng & Milad, 2015).

Likewise, traumatic events can influence gonadal systems, further strengthening evidence that there is a bidirectional relationship between gonadal systems and trauma disorders. Early childhood trauma has been found to affect the HPG axis; childhood trauma is linked with early puberty in females but not males; and rapid progression through puberty is linked to an increased risk of developing a depressive disorder in females but not males (Kuehner, 2017). Physiological stress response mediated by the HPA axis is altered by menarche, pregnancy, menopause, stages of the menstrual cycle, and the oral contraceptive pill (Kajantie & Phillips, 2006). This relationship implies that gonadal hormones mediate the effect a trauma has on the development of a mental illness. This interaction between trauma, hormonal development, and psychiatric vulnerability may also help explain why early adversity appears to disproportionately predispose women to FND.

Sex hormones have been implicated in the pathogenesis and effect treatment response in psychosis. Being postmenopausal is a negative predictor of treatment response in females with schizophrenia and increases the likelihood of resistance to antipsychotics. Raloxifene, a SORM, and estrogen have been demonstrated to augment antipsychotic efficacy in postmenopausal women (Gonzalez-Herrero, Morgante, Pagonabarraga, Stanton, & Edwards, 2022; Kulkarni, 2023). One of the proposed pathophysiological processes for schizophrenia is abnormal synaptic pruning (Boksa, 2012) and estrogen has been linked with delayed dendritic pruning, while testosterone has been linked with enhanced pruning (Savic, 2014). This could possibly contribute to schizophrenia occurring earlier in males than females.

Further implicating the role of gonadal hormones is that the gender ratio in some psychiatric conditions reverses before and after puberty. Neuroticism is present equally in females and males in childhood but greatly increases in females compared to males during adolescence (Kuehner, 2017). OCD is more common in women than men (1.6:1) over a lifetime, but prior to puberty it is far more common in boys than girls (3:1) (McLean & Anderson, 2009). It is unclear whether this is more influenced by biological or sociological factors, however the difference is more pronounced in developed countries, which implies a prominent environmental influence (Kuehner, 2017).

Like other mental illnesses, generalized anxiety disorder is influenced by the menstrual cycle, pregnancy, the postnatal and perimenopausal periods, implying the possible influence of gonadal hormones (Hantsoo & Epperson, 2017; Jalnapurkar, Allen, & Pigott, 2018). However, there is evidence of an interaction of psychosocial with biological factors, as the development of generalized anxiety disorder in pregnancy is influenced by associated factors such as: a previous history of generalized anxiety disorder, a lower level of education, and a history of childhood abuse (Jalnapurkar et al., 2018).

### Psychosocial factors

Gender-based discrimination, such as in economic position, access to resources and social status, is believed by some to be the largest contributor to the gender imbalance seen across mental illnesses overrepresented in women (Kuehner, 2017). This occurs not just through causing more adversity, contributing to the pathogenesis of illness but also reducing access to treatment through socioeconomic power, potentially prolonging psychiatric conditions (McLoughlin et al., 2023).

Gender-based violence has a serious effect on mental and physical health (Kuehner, 2017), as does childhood sexual abuse, which disproportionately affects girls, and has been linked to the development of a dysfunctional endocrine stress response, dysfunctions in autoimmune processes, structural and functional brain changes, reduced telomerase length, and altered DNA methylation (Kuehner, 2017).

Masculine and feminine gender norms can affect the likelihood of an individual developing a psychiatric illness. Traditional ideas of masculinity, such as believing that men must be self-reliant, tough, dominant, and suppress their emotions, are associated with an increase in PTSD, possibly through the inhibition of processing of traumatic events. However, other notions of masculinity (such as dedicated to pursuing success) can reduce the risks of developing PTSD (Christiansen & Berke, 2020). Changing social norms are resulting in a reduction in the gender ratio in eating disorders (Mitchison, Hay, Slewa-Younan, & Mond, 2014).

Gender norms can also affect help-seeking behaviors, and thus diagnosis rates. The artefact hypothesis proposes that one gender may be less likely to be diagnosed with an illness due to bias influencing those making the diagnosis, or through different help-seeking behaviors between men and women (Kuehner, 2017; McLoughlin et al., 2023). Biases in those making the diagnosis and differences in presentation have been suggested as reasons for the stark gender ratio in autism (Posserud et al., 2021). This is possibly due to the diagnostic criteria being based on a phenotypic presentation typical for boys with autism. The gender ratio has been found to be larger in people who present with social difficulties without an intellectual disability (Posserud et al., 2021). Women with anxiety disorders are more likely to present with somatic symptoms than men, which has been proposed to be secondary to the influence of social factors making women more prone to internalizing disorders than men (Jalnapurkar et al., 2018). This may result in women being more likely to be diagnosed with anxiety disorders.

## Explanations for gender differences seen in neurological conditions

### Biological factors

Female gonadal hormones have been implicated in increasing migraine severity. Estrogen has a clear association with worse migraine outcomes, as high estrogen levels, high fluctuations in estrogen, and hormone replacement therapy are all associated with worse migraines (Amiri et al., 2021). However, some migraine types are associated with menstruation, implying the role of estrogen and progesterone, or lack thereof, in their pathogenesis (Victor, Hu, Campbell, Buse, & Lipton, 2010). One potential explanation for menstrual migraines is that estrogen increases glutamatergic and serotonergic tone in the trigeminal nucleus caudalis, and progesterone causes the opposite (Allais et al., 2020). Estrogen and progesterone can also raise CGRP production, which modulates pain, making people with higher estrogen levels more prone to experiencing pain (Allais et al., 2020). Given that heightened pain sensitivity is also reported in FND, similar hormone-driven mechanisms may contribute to the disorder’s female predominance (McLoughlin et al., 2023).

Epilepsy has a complex relationship with estrogen. Estradiol increases glutamatergic transmission, making it a proconvulsant (Pottoo et al., 2019). However, some women with primary generalized epilepsy experience fewer seizures at the stage of their menstrual cycle when estrogen is the highest, and in some cases estrogen replacement therapy has reduced seizures (Veliskova, De Jesus, Kaur, & Velisek, 2010). The oral contraceptive pill has not been linked with changes to seizures (Veliskova et al., 2010). Β-estradiol, which has a neuroprotective effect (Pottoo et al., 2019), may, with chronic administration, be protective for seizures (Veliskova et al., 2010). These hormonal influences on seizure activity may be relevant for functional seizures, where altered excitability could lower the threshold for symptom manifestation.

There are multiple indicators that estrogen plays a role in the pathogenesis of MS, including the increase in risk of developing MS in females after puberty, earlier puberty being a risk factor for females developing MS, and changes to relapse risks during the third trimester of pregnancy (Bove & Chitnis, 2014). That reproductive hormonal shifts can alter MS risk suggests that similar mechanisms could also influence symptom onset or exacerbation in FND, particularly during pregnancy or postpartum periods.

AD is also affected by sex hormones. The ε4 allele of the apolipoprotein ε gene is associated with early onset of AD in females but not in males (Mielke et al., 2014). This is also true of the Met66 allele of brain-derived neurotrophic factor; conversely, there are genes that affect males’ chances of developing AD but not females’ (Mielke et al., 2014). Menopause imposes a change in gonadal hormones that is not experienced by males, and may be relevant to the development of neurological conditions later in life. Females experience a rapid loss of estrogen, progesterone, testosterone, and a disruption of the HPG axis (Mielke et al., 2014). Whilst males experience a decline in testosterone (which is metabolized to estrogen) with age, the decline is gradual (2–3% a year after 30) (Mielke et al., 2014). It may be that females are more likely to develop AD due to lower levels of estrogen, which has a demonstrated neuroprotective effect. Observational studies have shown that hormone replacement therapy is associated with lower levels of AD, however, the timing appears to be important (hormone therapy commenced within 5 years of menopause is protective) (Mielke et al., 2014). This highlights how menopausal hormonal changes might also be relevant to FND, in keeping with the changes in prevalence across people’s lifespan.

Not only are males more likely to develop PD than females, but females who develop PD are more likely to have a benign illness (at least at first) (Picillo et al., 2017). It appears that the length of estrogen exposure is the factor responsible for this difference, as estrogen is believed to be protective against dopaminergic neurological damage (Picillo et al., 2017). Although the mechanisms differ, this illustrates how estrogen modulates neurological vulnerability, offering a parallel for the sex differences in FND.

### Psychosocial factors

The gender ratio in MS has changed over the past 80 years, due to the increased incidence of relapsing remitting MS, which primarily affects women (Trojano et al., 2012). It is believed that the rise in relapsing remitting MS is secondary to women spending more time indoors due to urbanization, increased education and participation in the workforce, increased smoking, and rising levels of obesity (Bove & Chitnis, 2014; Tamaddon et al., 2021).

Diseases associated with advanced age, such as AD, may be more common in women as they live longer, and are thus more likely to contract age-related illnesses (Mielke et al., 2014). However, behavior associated with feminine gender norms such as reduced rates of smoking, higher educational attainment, and increased likelihood to exercise later in life, are all protective for AD (Mielke et al., 2014).

It is possible that misdiagnosis of FND, due to diagnostic biases, has resulted in a smaller gender ratio in MS and a larger gender ratio in PD, due to men being more likely to be diagnosed with MS and PD than FND. However, misdiagnosis of FND is rare and the effect is likely minimal (McLoughlin et al., 2023).

## Relevance of gender ratios across disorders to FND

Across psychiatric and neurological illnesses, gender ratios follow a consistent pattern. Conditions characterized by internalizing symptoms, histories of trauma, and dissociation (e.g. depression (Kuehner, 2017), anxiety disorders (McLean & Anderson, 2009), PTSD (Christiansen & Berke, 2020), eating disorders (Qian et al., 2022), and migraines (Victor et al., 2010)) are more common in women. By contrast, male predominance is seen in neurodevelopmental (e.g. autism (Posserud et al., 2021), externalizing conditions (substance use disorder (Greenfield, Back, Lawson, & Brady, 2010)), and schizophrenia (Abel et al., 2010)). Looking at gender ratios through this lens, FND sits at the strongly female predominant end of the spectrum, second only to anorexia nervosa among the conditions we assessed. This position suggests that FND is better understood alongside other internalizing, trauma-related and dissociative conditions, rather than as an atypical neurological disorder, and supports a hypothesis that its gender imbalance arises through hormone/stress interactions (stress-diathesis model), which is exacerbated by gendered social adversity.

### Similarities with eating disorders

FND and eating disorders share multiple demographic, clinical, and psychosocial features. Both are strongly female-predominant, often emerge in adolescence or early adulthood, and are associated with trauma (similar proportion of patients reporting a precipitating traumatic event within 6 months prior to onset of symptoms), alexithymia, and dissociation (Demartini et al., 2017). Anorexia and FND share similar marital status (the majority are single) and educational attainment (most have completed high school) (Demartini et al., 2017). Binge eating disorder, the eating disorder with the least pronounced gender ratio (1:2.1), has been associated with functional seizures (Mammi et al., 2024). Both FND and anorexia are associated with autism, which may explain these common factors (Brede et al., 2020; Gonzalez-Herrero et al., 2022).

### Biological factors

Biological sensitivity to gonadal hormones may also help explain gender differences in FND. Estrogen and progesterone fluctuations across menarche, the menstrual cycle, and menopause, affect brain regions relevant to agency and stress reactivity, influencing the likelihood of the development of an internalizing disorder. The right temporo-parietal junction (rTPJ) has been shown to be hypoactivated in FND (Baizabal-Carvallo, Hallett, & Jankovic, 2019) and plays a key role in the sense of agency, which is impaired in FND (Zito, Wiest, & Aybek, 2020). It is influenced by estrogen levels, with rTPJ volume and functional connectivity correlating with estrogen levels (De Bondt, Pullens, Van Hecke, Jacquemyn, & Parizel, 2016; Hidalgo-Lopez et al., 2020). Estrogen and progesterone also modulate neuronal excitability and neurotransmitter systems, influencing pain perception and seizure propensity (Allais et al., 2020; Pottoo et al., 2019). These mechanisms could be relevant in FND by altering the sense of agency, and impacting functional seizures and somatic pain symptoms.

The differing impact of early childhood trauma childhood on the genders may have implications for FND. Trauma in girls leads to alteration of the HPG axis, causing early puberty and increased risk of depression (Kuehner, 2017; Mu et al., 2022), but this effect is not found in boys. The anxiolytic effect of testosterone (Christiansen & Berke, 2020) may mean that males are less likely to develop FND in response to adversity later in life; while estrogen’s impact on fear responses may mean that females are more likely to develop FND in response to the same adversities (Maeng & Milad, 2015). Thus, the interplay of trauma, hormonal axes, and stress responses further explains why women face a heightened vulnerability to FND following adverse experiences. These differences are exacerbated by childhood trauma occurring in girls more frequently than boys. It may be that HPA dysfunction is the biological modulator of stress responses, as postulated by the stress-diathesis model (Keynejad et al., 2019).

Women are more prone to internalizing disorders (e.g. depression, anxiety, and somatization), which have been linked with reduced release of cortisol in response to stress in females than in males (Hartman, Hermanns, de Jong, & Ormel, 2013). Fluctuations in estrogen and progesterone are also linked with an increased risk of internalizing disorders, as is evidenced by the effects of the menstrual cycle, pregnancy, and menopause on these conditions (Karpinski et al., 2017; Mu et al., 2022). MS is also influenced by pregnancy (Bove & Chitnis, 2014). Like internalizing disorders and MS, there is evidence at the case study level that FND can be affected by pregnancy (Cabreira et al., 2024). Given this, it is reasonable to expect that fluctuations in estrogen and progesterone could increase the risk of someone developing FND.

### Psychosocial factors

The difference in propensity to internalizing disorders may also be secondary to the increased stress placed on women from discrimination and societal inequality, higher levels of abuse, stress from gender-role related societal expectations, gender-role related help-seeking behavior, or bias in those diagnosing these conditions contributing to women’s risk of developing them (Jalnapurkar et al., 2018; Kuehner, 2017; McLoughlin et al., 2023).

Economic disparities, access to resources, and inequality in social status between genders are major contributors to the gender imbalance in mental illnesses (McLoughlin et al., 2023). Women’s relatively reduced socioeconomic power directly adversely affects their health, and results in reduced access to mental health care, potentially prolonging their psychiatric, neurological, or neuropsychiatric conditions (McLoughlin et al., 2023). Gender-based violence and childhood sexual abuse, which disproportionately effects women, has profound effects on both mental and physical health (Kuehner, 2017).

Gender norms also play a pivotal role in shaping differential risks and presentations of mental illnesses. Traditional gender norms, have been suggested to increase PTSD risk in men (due to the suppression of emotions) (Christiansen & Berke, 2020), be protective of illness in women (through reduced smoking rates, higher education attainment, and a tendency to exercise later in life) (Mielke et al., 2014) and affect help-seeking behaviors (women are more likely to seek help for certain conditions leading to diagnostic biases) (Kuehner, 2017; McLoughlin et al., 2023). Gender norms affecting help-seeking behavior may result in an artefact hypothesis in FND, with women being more likely to seek help for somatic symptoms than men. In this way, gender norms shape who receives care, potentially skewing understanding of the gender ratio of FND.

### Integration

The stress-diathesis model attempts to explain the complexities in pathogenesis by postulating that there are biological risks that are mediated by psychological and social factors resulting in FND (Keynejad et al., 2019). This proposed mechanism is unidirectional, with biological risks existing from birth being mediated by psychological and social factors emerging from the post-natal environment. However, it fails to capture the bidirectional nature of the relationships between factors, e.g. childhood sexual assault (which is more likely to affect girls due to societal structure), results in girls (but not boys) being more likely to undergo early puberty, increasing the risk of them developing a depressive disorder, whilst early puberty does not carry the same risk of later depression in boys. Given the complexity and interplay of these factors, disentangling the biological from the psychosocial in FND could ultimately be impossible.

In FND, women are more likely exposed to trauma due to socioeconomic and cultural inequalities and gender-based violence (Kuehner, 2017; McLoughlin et al., 2023). This adversity biologically impacts their stress-response systems (HPA and HPG axes), exacerbating vulnerability to neurological manifestations such as dissociation and loss of agency. Concurrently, societal expectations of femininity, including expression of somatic symptoms, enhance symptom severity and affect diagnostic bias.

### Strengths and limitations

This review drew on three major databases spanning psychiatry, psychology and neurology, and integrated literature from both psychiatric and neurological disorders to situate FND within a broader gendered landscape. It synthesized evidence from multiple potential mechanisms, generating testable hypotheses for future empirical work.

However, important limitations should be noted. First, this was a narrative rather than a systematic review, so the search and selection process may have missed relevant studies and is more vulnerable to selection bias than a formal systematic review. Second, the included studies were highly heterogeneous in terms of diagnostic criteria, recruitment methods and clinical settings, limiting the comparability of gender ratios across disorders and precluding meta-analytic synthesis. Third, it relied largely on published, English-language peer-reviewed literature from MEDLINE, PsycINFO, and Web of Science, which may introduce publication and language bias and under-represent non-Western populations. Fourth, most of the available data report binary sex/gender categories, so our conclusions mainly concern women versus men and cannot adequately address gender-diverse populations. Fifth, it may be that the disorders examined were not extensive enough and if other neurological and psychiatric conditions were included the results may have been different. Sixth, the study was limited to medical research, which may be biased in not considering psychosocial causes in as much depth as biological causes. Finally, as clinicians and researchers with a particular interest in FND, our selection and interpretation of the literature may have been influenced by our prior conceptual models; we have attempted to mitigate this by explicitly outlining our reasoning and highlighting areas of uncertainty.

## Conclusion

This review looked at gender in FND through the novel lens of the gender ratios in other neurological and psychiatric conditions. It found that, compared to the gender ratios across other psychiatric and neurological disorders, FND is one of the most female-predominant conditions. Its gender ratio suggests that FND is better understood alongside other internalizing, trauma-linked and dissociative conditions, such as eating disorders, rather than as a typical neurological disorder. We propose a model in which gonadal hormones interact with early-life trauma and gendered social adversity to shape vulnerability to FND, through effects on stress-response systems and neural circuits supporting agency and interoception. This is an adjustment to the existing stress-diathesis model in that a primary modulator of diathesis is gonadal hormones and the relationship is bi-directional, where the stress modulates the and the hormones modulate the impact of the stress. Understanding how these intersecting factors interact could guide future research and treatment.
